# Fear Spreading Across Senses: Visual Emotional Events Alter Cortical Responses to Touch, Audition, and Vision

**DOI:** 10.1093/cercor/bhw337

**Published:** 2016-11-22

**Authors:** Judith Domínguez-Borràs, Sebastian Walter Rieger, Corrado Corradi-Dell'Acqua, Rémi Neveu, Patrik Vuilleumier

**Affiliations:** 1 Laboratory for Behavioral Neurology and Imaging of Cognition, Department of Neuroscience, University Medical Center, CH-1211 Geneva, Switzerland; 2 Swiss Center for Affective Sciences, University of Geneva, Campus Biotech, CH-1202 Geneva, Switzerland; 3 Geneva Neuroscience Center, University of Geneva, CH-1211 Geneva, Switzerland; 4 Department of Psychology, FPSE, University of Geneva, CH-1205, Geneva, Switzerland; 5 Department of Neurology, University Hospital, CH-1211 Geneva, Switzerland

**Keywords:** attention, emotion, fMRI, sensory modulation

## Abstract

Attention and perception are potentiated for emotionally significant stimuli, promoting efficient reactivity and survival. But does such enhancement extend to stimuli simultaneously presented across different sensory modalities? We used functional magnetic resonance imaging in humans to examine the effects of visual emotional signals on concomitant sensory inputs in auditory, somatosensory, and visual modalities. First, we identified sensory areas responsive to task-irrelevant tones, touches, or flickers, presented bilaterally while participants attended to either a neutral or a fearful face. Then, we measured whether these responses were modulated by the emotional content of the face. Sensory responses in primary cortices were enhanced for auditory and tactile stimuli when these appeared with fearful faces, compared with neutral, but striate cortex responses to the visual stimuli were reduced in the left hemisphere, plausibly as a consequence of sensory competition. Finally, conjunction and functional connectivity analyses identified 2 distinct networks presumably responsible for these emotional modulatory processes, involving cingulate, insular, and orbitofrontal cortices for the increased sensory responses, and ventrolateral prefrontal cortex for the decreased sensory responses. These results suggest that emotion tunes the excitability of sensory systems across multiple modalities simultaneously, allowing the individual to adaptively process incoming inputs in a potentially threatening environment.

## Introduction

Survival requires efficient detection of salient information in an overwhelming sensory environment. It is well established that the human brain responds more rapidly (Flykt and Caldara 2006; Yang et al. 2007; Gerritsen et al. 2008; Amting et al. 2010) and shows stronger activation of early sensory regions to emotionally significant than to neutral stimuli, such as violent scenes, fearful faces, or angry voices (Lang et al. 1998; Vuilleumier et al. 2001; Pourtois et al. 2004a; Grandjean et al. 2005; Vuilleumier 2005; Lim et al. 2009; Sabatinelli et al. 2011; Domínguez-Borràs and Vuilleumier 2013). However, it remains unclear whether these modulatory effects of emotion may also take place across different sensory modalities, as in real life the brain is confronted with multiple simultaneous sensory inputs through distinct afferent pathways. For instance, spatial attention is widely known to exert crossmodal influences, modulating early sensory analysis across the visual, auditory, and somatosensory cortices (Calvert 2001; Eimer and Driver 2001; McDonald et al. 2003; Busse et al. 2005; Spence 2010; Wesslein et al. 2014).

In the emotion domain, it has been suggested that threatening contexts may sensitize reactivity to stimuli in all sensory modalities, sometimes at a loss of stimulus specificity (Baas et al. 2006; Cornwell et al. 2007; Dunning et al. 2013; Sharvit et al. 2016). Recent research using electrophysiology and functional magnetic resonance imaging (fMRI) suggests that unattended sounds elicit stronger brain responses when participants are concurrently viewing emotionally negative, as compared with neutral images (Sugimoto et al. 2007; Domínguez-Borràs et al. 2008a, 2008b, 2009; Garcia-Garcia et al. 2010; Selinger et al. 2013). However, to our knowledge, no study previously examined whether this affective potentiation of sensory processing may extend between modalities, and whether it may rely on common neural mechanisms across senses.

Here, we tested whether visual emotional signals affect sensory responses to unattended stimuli across 3 sensory modalities in a single fMRI experiment. To this end, we measured how the emotional content of a visual stimulus impacted on the processing of concurrent inputs in the auditory, tactile, and visual modalities. While participants attended to an emotionally neutral or fearful face in the center of the screen and performed a gender categorization task, they were presented with bilateral task-irrelevant tones, tactile stimuli on the cheek, or flickering checkerboards.

We then compared fMRI responses in auditory, somatosensory, and visual cortices evoked by the task-irrelevant stimuli in both emotion conditions. If visual emotional events facilitate the processing of task-irrelevant stimuli across modalities (see Domínguez-Borràs et al. 2008a, 2008b, 2009), the corresponding sensory areas should become more responsive. If emotional processing results in sensory depletion due to competitive allocation of resources between modalities (Pessoa et al. 2003), sensory areas should exhibit lower responses instead. In addition, we performed conjunction and connectivity analyses to uncover any common network that might provide emotional modulatory signals to the different sensory modalities. Our results bring new insights into how emotion processing interacts with elementary sensory processing across modalities.

## Materials and Methods

### Subjects

Because emotional processing is often associated with gender differences (Kret and De Gelder 2012) and previous research showed that females exhibit stronger crossmodal effects for visual emotional information (Garcia-Garcia et al. 2008), we chose to recruit female participants only (*N* = 19; age 18–33 years, mean 22.21 ± 4.09 years) as in prior experiments (Domínguez-Borràs et al. 2009). All participants were right-handed, with reported normal hearing, normal or corrected-to-normal vision, and normal tactile sensation. None of them had any history of neurological or psychiatric illness. The study was approved by the local ethics committee and conducted according to the declaration of Helsinki. All participants gave written informed consent and completed the State-Trait Anxiety Inventory (Spielberger et al. 1983), which revealed anxiety scores within the normal range in all cases (STAI-State: *M* = 41 vs 38.8 in standard population, SD = 4; STAI-Trait: *M* = 46.52 vs 40.4 in standard population, SD = 7.22).

### Stimuli

#### Emotional Stimuli

The target stimuli consisted of 52 black and white pictures of faces from the NimStim (Tottenham et al. 2009) and the Karolinska Directed Emotional Faces (Lundqvist et al. 1998) databases, including 13 male and 13 female actors, each of them shown with either a neutral or a fearful expression. Emotional faces provide a reliable tool to recruit emotion processing systems in humans (Hariri et al. 2002; Vuilleumier and Pourtois 2007; Sabatinelli et al. 2011; Ahs et al. 2014). Please note that the term “emotion” here should not be taken as synonymous with “feelings” or “emotional experience” (see LeDoux 2012), but as an involuntary sequence of neural and behavioral responses derived from the processing of biologically relevant stimuli, including enhanced activation of sensory areas or amygdala (see Pourtois et al. 2012 for a review).

A further 6 faces, with happy expression, were used for an additional filler block (unanalyzed). The luminance of all images was standardized. Pictures had a resolution of 406 × 525 pixels, corresponding to 6.2° × 8° of visual angle. Each face stimulus appeared 6 times.

#### Task-Irrelevant Auditory Stimuli

The auditory stimuli consisted of a complex tone (Aud), presented binaurally, with a fundamental frequency of 500 Hz and 2 harmonics of 1000 Hz and 1500 Hz (fade-in: 5 ms; fade-out: 20 ms; duration: 100 ms). The intensity of each subsequent harmonic tone was 50% of the intensity of the fundamental tone (for intensity, see Procedure). The aim of using complex tones was to improve robustness of the group blood oxygenation level dependent (BOLD)-associated responses by stimulating a more extensive area along the tonotopic auditory cortex. This procedure was considered preferable as compared with pure tones or white noise (the latter also being a potential source of unwanted startle responses), and it followed the same methodology as that used in a previous study of ours (Selinger et al. 2013).

#### Task-Irrelevant Tactile Stimuli

**Figure 1.**
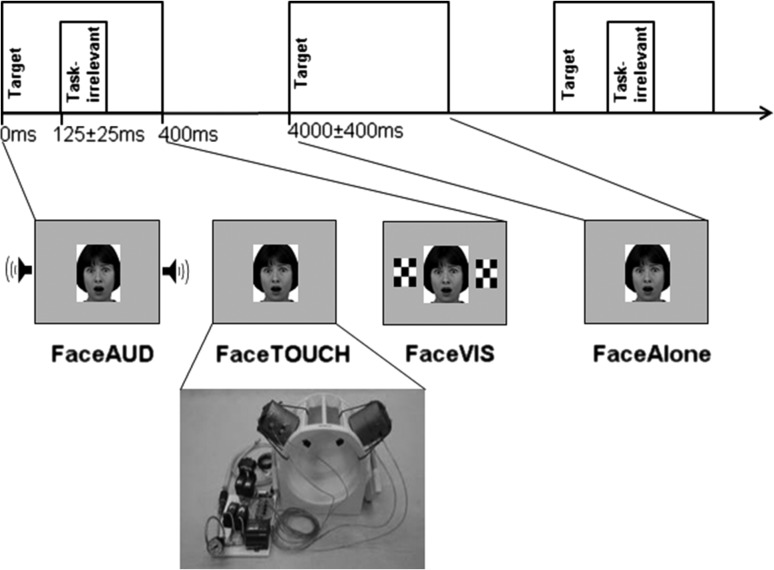
Trial structure. Participants judged the gender of a face (the target), which could be either neutral (NEU condition) or fearful (NEG condition). Faces were presented either alone (FaceAlone condition) or concomitantly with one of the three task-irrelevant sensory stimuli, which subjects were instructed to ignore. These consisted of either a binaural complex tone (FaceAud condition), a single nonpainful tap applied to both lower cheeks (FaceTouch condition), or 2 flickering checkerboards on screen (FaceVis condition).

The tactile stimuli consisted of a single nonpainful tap of 100 ms duration (Touch) and were applied to both lower cheeks of the participant (in order to match the subjective proximity of the other task-irrelevant stimuli with respect to the personal space). These were delivered with a custom-built device comprising nonmetallic pneumatic cylinders (TA-AC-PVC-1.0-EP, Teqcom Industries, Inc.; see Fig. 1). The cylinders were controlled by electromagnetic valves, which were placed outside the MRI scanner room and connected to a programmable control unit. The cylinders were in permanent contact with the skin and traveled forward by 5 mm when activated.

#### Task-Irrelevant Visual Stimuli

The visual stimuli consisted of 2 flickering checkerboards (Vis), presented on either side of the face image (150 × 150 pixels, corresponding to 11.18° × 2.28° visual angle; 30 Hz; duration 100 ms).

### Task and Conditions

The experimental task was delivered with Cogent 2000 v1.32 (Wellcome Department of Imaging Neuroscience, London, UK) running on Matlab R2009b. Participants lay supine inside the MRI scanner and saw a monitor through a mirror mounted on the MR head coil. In each trial, one face, which could be neutral (NEU condition) or fearful (NEG condition), was presented for 400 ms at the center of the screen, on a light gray background. Subjects were instructed to press a response button with their right hand (index or middle finger, counterbalanced across subjects) to indicate whether it depicted a male or a female.

Faces were either presented alone (FaceAlone condition) or concomitantly with one of the three task-irrelevant stimuli (which the subjects had been instructed to ignore), resulting in the conditions FaceAud, FaceTouch, and FaceVis, respectively (Fig. 1). In order to avoid oddball-like responses triggered by a smaller proportion of trials without task-irrelevant stimulation (Naatanen et al. 2011), the FaceAlone trials represented 50% of the experimental trials, and trials with task-irrelevant stimuli represented the other 50%. Task-irrelevant stimuli were delivered 125 ± 25 ms after face onset, as this stimulus asynchrony was revealed to be optimal in a previous study examining crossmodal visual-auditory emotion effects (Selinger et al. 2013). Trial length ranged from 3600 to 4400 ms (mean 4000 ± 400 ms). A single sequence was designed, within which 312 trials were distributed into pseudorandom series of 3, 4, 5, 6, or 7 trials where faces displaying the same emotional expression were presented successively (counterbalanced across subjects). This procedure allowed us to maximize the fMRI signal, as the shortest continuous scanning period per emotional condition lasted 11 s (3 trials of 3600 ms), with 65 NEU to NEG or NEG to NEU transitions. The presentation of task-irrelevant stimulation (or its absence for the FaceAlone condition), however, was completely randomized within the sequences. An additional series of 78 trials with no stimulation at all (NullTrials; mean length 4000 ms) was randomly interspersed with the experimental trials, so as to avoid saturation of the fMRI signal (but these null trials were not included in the analysis).

### Procedure

Before each measurement, hearing threshold was determined inside the scanner while an echo-planar sequence was run. The same complex tone that would be later used for the experiment was presented repeatedly to the subject through both ears, first, at the lowest possible volume, then at progressively higher volume until the subject reported hearing it above the scanner noise (please note that sound intensity was never reportedly too loud or painful for the participant). Volume manipulation was done through an amplifier from the MRI control room. Finally, the sound volume was set to 40 dB above the determined threshold. Right-left balance was then adjusted, if necessary, until participants reported equal perceived volume on either side. Adjustment and tests of the somatosensory stimulation device were also performed to ensure correct functioning, symmetric stimulation on both cheeks and comfort for each participant.

Pupillary size was measured continuously with an eye-tracking system at 60 Hz (EyeTrac 6, Applied Science Laboratories). This measure allowed us to estimate autonomic arousal levels (Critchley et al. 2005; Sterpenich et al. 2006). At the end of the experimental session, subjects rated the faces previously seen for valence and arousal on a continuous 1–11 scale, where 1 was labeled on the screen as “very positive” or “very relaxing,” 6 as “neutral,” and 11 as either “very negative” or “very alerting.”

### Data Acquisition and Preprocessing

The MRI data were acquired on a 3T whole body MRI scanner (Trio TIM, Siemens), using a 12-channel head coil. Structural images were acquired with a T1-weighted 3D sequence (MPRAGE, TR 1900 ms, TE 2.27 ms, TI 900 ms, flip angle 9°, 256 × 256 × 192 voxels, 1 mm isotropic). Functional images were obtained using a susceptibility weighted echo-planar (EPI) sequence optimized for BOLD contrast (TR 2100 ms, TE 30 ms, flip angle 80°, 36 slices of 64 × 64 voxels, 3.2 mm isotropic, 20% slice gap, sequential descending slice order, PAT factor 2). Image processing was carried out using SPM8 (http://www.fil.ion.ucl.ac.uk/spm/), images were realigned, slice-time corrected, and coregistered with the T1 anatomical image. The anatomical images were normalized to the Montreal Neurological Institute (MNI) single-subject template using the “unified segmentation” function in SPM8. This algorithm is based on a probabilistic framework that enables image registration, tissue classification, and bias correction to be combined within the same generative model (Ashburner and Friston 2005). The resulting deformation fields were then used for normalization of all individual functional images to MNI space. Finally, the images were resampled to an isotropic voxel size of 2 mm and spatially smoothed with an 8-mm FWHM Gaussian kernel to compensate for residual macroanatomical variations across subjects (Friston et al. 1995).

### Data Analysis

Behavioral performance was analyzed, on one hand, by calculating accuracy (hit rate; HR) and hit response time (Hit-RT; i.e., latencies of correct response) for every condition. On the other hand, we also estimated distraction/facilitation effects due to the task-irrelevant stimuli, by subtracting Hit-RT in FaceAlone conditions from Hit-RT in FaceAud, FaceTouch, and FaceVis conditions. Data were then compared by means of a 2-factor repeated-measure ANOVA (using the 2 factors “Task-irrelevant stimulus”: FaceAud, FaceTouch, FaceVis and “Emotion”: NEU, NEG) with Greenhouse-Geisser adjustments to the degrees of freedom. Post hoc comparisons were performed using the Bonferroni adjustment for multiple comparisons.

The fMRI data were analyzed using the general linear model (GLM) framework implemented in SPM8 (Kiebel and Holmes 2004). At the first level, 9 regressors were included in the statistical model: 8 corresponding to our experimental conditions and 1 for trials with misses or errors. To account for movement-related variance, we included 6 nuisance regressors representing the differential of the 6 movement parameters from the realignment. Data were high-pass filtered (1/128 Hz), corrected for intrinsic autocorrelations, and convolved with a standard HRF. We accounted for putative habituation effects in neural responses by modeling a linear time-dependent modulation that creates, for each condition, an additional regressor in which the BOLD response amplitude was modulated parametrically according to trial order across the whole experimental session.

A second-level flexible factorial analysis (random-effects) was performed on the single-subject parameter estimate maps associated with the 8 main conditions of interest, with "conditions" as the within-subject factor and "subjects" as random factor. To test for putative effects associated with habituation, an additional flexible factorial analysis was run using the parameter estimates associated with parametric effect of time (trial order).

Within each of these 2 flexible factorial models, 7 contrasts of interest were computed, using voxel-by-voxel *t*-tests, to assess for differential effects across the following conditions: FaceAloneNEG vs FaceAloneNEU: “main effect of emotional face”; FaceAudNEU vs FaceAloneNEU: “auditory processing in neutral condition”; FaceAudNEG vs FaceAloneNEG: “auditory processing in negative condition”; FaceTouchNEU vs FaceAloneNEU: “tactile processing in neutral condition”; FaceTouchNEG vs FaceAloneNEG: “tactile processing in negative condition”; FaceVisNEU vs FaceAloneNEU: “visual processing in neutral condition”; FaceVisNEG vs FaceAloneNEG: “visual processing in negative condition.” These contrasts of interest compared NEU versus NEG conditions in both directions (> and <), in order to examine the modulatory effects of emotion on task-irrelevant sensory responses to distractors (i.e., the interaction between emotion and distractor effects: [{FaceAudNEG > FaceAloneNEG} > {FaceAudNEU > FaceAloneNEU}], and so on). The conditions with task-irrelevant stimulation were also compared with the FaceAlone conditions (pooling NEU and NEG expressions together; i.e., [{FaceAudNEU + FaceAudNEG} > {FaceAloneNEU+FaceAloneNEG}]) to determine task-irrelevant responses in each sensory modality (see below).

Conjunction analysis was also performed to identify any common brain activation for all 3 sensory modalities, excluding significant voxels derived from the main effect of “emotional faces themselves, i.e., when presented alone” (with an exclusive mask from the latter contrast at *P* < 0.05 uncorrected). Conjunction was computed using the Global Null hypothesis (Friston et al. 1999). Since this approach may have weaknesses (e.g., Nichols et al. 2005) and potentially lead to results driven by only a few (not all) of the contrasts of interest, we applied an additional inclusive mask (with threshold of *P* < 0.05 uncorrected) for each of the 3 contrasts contributing to the conjunction (i.e., main effect of distractors in each sensory modality). This inclusive mask was combined with the exclusive mask from the “emotional face effect” mentioned above using the ImCalc function in SPM. To implement this conjunction analysis, we built a separate GLM model, including the regressors representing the 3 contrasts associated with the main modulatory effects (FaceAudNEG [time] > FaceAudNEU [time] ∩ FaceTouchNEG > FaceTouchNEU ∩ FaceVisNEG > FaceVisNEU).

We report activations exceeding a cluster-level threshold corresponding to *P* < 0.05, corrected for multiple comparisons for the whole brain (Friston et al. 1994), with an underlying height threshold corresponding to *P* < 0.001 (uncorrected). We also applied small-volume correction (Worsley et al. 1996) for a priori regions of interest (ROIs) as defined by anatomical masks. Within each of these ROIs, we considered reliable activations whose effects surviving small-volume family-wise error correction at the voxel level. All parametric maps were rendered on the average T1-weighted template of the whole group.

#### ROI Definition

When necessary, we constrained our hypothesis on a priori defined sensory-specific ROIs: for audition, primary auditory cortex (PAC, comprising Te1.0, Te1.1, and Te1.2); for touch, primary somatosensory cortex (S1); for vision, primary visual cortex (V1). An additional ROI mask for the amygdala was generated according to the hypothesis that this structure is critically involved in the emotional modulation of sensory processing (see Pourtois et al. 2012). These ROIs were based on standardized neuroanatomical divisions, independently defined by probabilistic cytoarchitectonic maps (i.e., Anatomy Toolbox for SPM), resulting from observer-independent quantification of cell volume densities and area borders, obtained from human postmortem brains (Eickhoff et al. 2005, 2007).

#### Controlling for Sensory-Specific Effects of Visual Emotion Processing

To verify whether any observed effects of visual emotional signals were genuinely explained by crossmodal sensory modulation (i.e., impact of emotional face processing onto the modality-specific cortices for the corresponding sensory stimuli in each condition) or rather by a more general, nonspecific modulation of all cortical areas (i.e., affecting even nonstimulated modalities), we also computed each of the main contrasts (listed above) for sensory ROIs that were not related to the current stimulus condition. For instance, we examined the contrast FaceAudNEG > FaceAudNEU on visual (V1) and somatosensory (S1) ROIs (using small-volume correction), and so on for the other modalities. Again, all ROIs were defined by masks from a standard atlas (see above).

#### Psycho-Physiological Interactions Analysis

To determine possible sources of the emotion-related sensory effects, we conducted a traditional psycho-physiological interaction (PPI) analysis (Friston et al. 1997). This method estimates functional connectivity via changes in inter-regional covariance as a function of different experimental manipulations or tasks. First, eigenvariate values were extracted for each participant from the filtered BOLD signal in the peaks with maximal *T* value for the 3 main contrasts of interest (i.e., those peaks showing the strongest emotion-related modulations in the group analysis) in each sensory modality (i.e., auditory, somatosensory, and visual cortices). Then, the time series were deconvolved to obtain an estimate of the event-related neural response, multiplied by the psychological condition of interest (FaceAudNEU, FaceAudNEG, FaceVisNEU, so on), and reconvolved using the canonical HRF to obtain a PPI regressor. We run three PPI models, one for each sensory modality contrast (Aud, Touch, and Vis). Each PPI model included a “Psychological task” regressor (FaceAudNEG vs FaceAudNEU, FaceTouchNEG vs FaceTouchNEU, FaceVisNEG vs FaceVisNEU, respectively); a “BOLD value” regressor, containing the signal extracted from the corresponding seed region; a PPI regressor, containing the interaction between the BOLD value and the Psychological task regressors; and our 6 nuisance regressors representing movement parameters. At the second level, we included the 3 PPIs of each of the 3 sensory modality contrasts in the same statistical model. The PPI regressors included for the Aud and Touch conditions reflected a positive correlation between the seed region (left PAC and left S1, respectively) and the event-related neural activity in other brain regions. Because activity in left V1 to task-irrelevant visual stimuli decreased in the NEG relative to the NEU emotional condition, for the visual condition, our model included the PPI regressor reflecting a negative correlation between the seed voxel (V1) and event-related activity in other brain regions. Finally, to identify common sources of modulation across modalities, we performed a second-level conjunction analysis across the 3 PPI contrasts of interest as follows: (FaceAudNEG > FaceAudNEU [positive correlation] ∩ FaceTouchNEG > FaceTouchNEU [positive correlation] ∩ FaceVisNEG > FaceVisNEU [negative correlation, which would be the same as FaceVisNEU > FaceVisNEG, positive correlation]). This analysis probed for any area showing significant changes in functional coupling (positive or negative, according to the concomitant emotion modulation of task-irrelevant sensory responses) across all 3 sensory modalities. Again, conjunction was computed by testing the Global Null hypothesis (Friston et al. 1999) with an additional inclusive mask (threshold corresponding to *P* < 0.05 uncorrected) for each of the 3 contrasts contributing to the conjunction.

#### Pupil Size Analysis

To obtain an independent physiological measure of emotional processing, pupillary size was analyzed for 15 subjects (data from 4 participants were lost due to technical reasons) by using Ilab (Gitelman 2002). Pupillary responses provide a reliable marker of emotional arousal (Sterpenich et al. 2006). Trials contaminated with blinks were discarded. Mean pupillary size was averaged over bins of 100 ms, for an epoch of 3 s for each trial, relative to a 500-ms prestimulus baseline. The analysis window ranged from 2000 to 3000 ms after face onset, following a period of pupil adaptation to changes in luminosity, in order to ensure that pupillary size would be stable enough to assess autonomic arousal (Sterpenich et al. 2006). Our analysis of interest focused on the FaceAloneNEU and FaceAloneNEG conditions, so as to assess pure autonomic responses elicited by the emotional face expression and to avoid confounds due to task-irrelevant stimulus responses, as well as to ensure comparability with the fMRI contrast main effect of emotional face (see above). We ran a linear mixed model in which we modeled pupil size as a function of “trial number” since the beginning of the experiment, “condition” (FaceAloneNEG vs. FaceAloneNEU), the interaction of both factors and “subject” as a random factor over the intercept, to take into account the repeated measures.

Additionally, for exploratory purposes only, we extracted pupillary size for all remaining conditions, and ran a new GLM analysis that included not only our nine original regressors (eight corresponding to our experimental conditions and one for trials with misses or errors) but also three additional parametric regressors accounting for variance according to pupil size, trial order, and interaction between these two covariates. The interaction regressor was created by multiplying both regressors after detrending them separately. In order to avoid the second and the third modulators to be serially orthogonalized by SPM with respect to previous modulators (which would make the corresponding beta estimate uninterpretable), we turned off the serial orthogonalization of parametric modulators hard-coded in SPM.

## Results

### Picture Rating

Average ratings by participants for the neutral and fearful faces, obtained in a postexperimental debrief session, were 6.13 ± 0.51 and 8.47 ± 0.7 for valence, respectively, and 5.55 ± 0.57 and 8.66 ± 0.63 for arousal, respectively. Therefore, fearful faces were reliably perceived as more negative and alerting than neutral faces (Valence: *T*_25_  = 15.140, *P* < 0.001; Arousal: *T*_25_ = 20.929, *P* < 0.001).

### Behavioral Data

Subjects performed almost flawlessly on the face gender task, with an average HR of ∼96% and average RT of 605 ms (see Table 1). There were no statistical differences in accuracy or Hit-RT between any of the conditions.

**Table 1:** Behavioral performance

	FaceAud	FaceVis	FaceTouch	FaceAlone
Hit rate (%)				
NEU	96.8 ± 3.69	96.36 ± 4.53	94.33 ± 6.2	96.29 ± 4.89
NEG	96.15 ± 4.06	96.76 ± 3.69	94.74 ± 7.5	95.95 ± 4.73
Response time (ms)				
NEU	591.59 ± 112.47	616.32 ± 126.9	605.4 ± 142.59	607.93 ± 129.45
NEG	596.68 ± 139.67	610.6 ± 152.2	604.63 ± 142.9	607.53 ± 126.25

Note: There were no statistical differences in accuracy or Hit response time between any of the conditions.

### Pupil Size

Face presentation induced systematic changes in pupillary size because of accommodation and light reflex evoked by the face onset and offset. After a relative stabilization of pupil size following stimulus offset, no significant difference in mean pupil size was observed for the FaceAloneNEG versus the FaceAloneNEU conditions (*T*_14_ = 0.212; *P* = 0.835). However, since faces were presented repeatedly, some habituation of arousal responses was expected (Plichta et al. 2014). A further analysis of variations across the course of the experiment revealed significant differences in pupil size between the FaceAloneNEG and the FaceAloneNEU conditions as a function of time (mean beta estimate for FaceAloneNEG vs. FaceAloneNEU: 0.198; SEM: 0.028; *t* = 7.079; *P* < 0.0001). Pupil size in FaceAloneNEG was larger than in FaceAloneNEU at the beginning of the experiment, but this difference disappeared (and even reversed polarity) toward the end of the experiment (slope for FaceAloneNEG: −0.091, *P* < 0.0001; slope for FaceAloneNEU: 0.106; *P* < 0.0001).

### Imaging Data

Tables 2–5 report all brain regions that, unless explicitly stated otherwise, survived rigorous correction for multiple comparisons either for the whole brain or for a priori defined ROIs.

**Table 2:** Processing of task-irrelevant stimuli (regardless of emotion condition)

Area	*xyz* coordinates			*T* value	size (voxels)	*P* value
Auditory (FaceAud > FaceAlone)						
R PAC (Heschl's gyrus)	56	−10	0	13.78	484	<0.0001**
	48	−18	−2	9.95		
L PAC (Heschl's gyrus)	−52	−22	2	14.3	5316	<0.0001*
R secondary auditory cortex (BA22)	58	−10	0	14.2	5319	<0.0001*
L secondary auditory cortex (BA42)	−64	−32	10	15.86	5316	<0.0001*
L medial geniculate nucleus	−14	−26	−6	5.16	18	0.0071*
L precentral gyrus (BA6)	−38	0	46	5.82	59	<0.0001*
L precuneus (BA7)	−6	−48	50	5.06	14	0.0096*
R precuneus (BA7)	6	−66	42	3.29	950	<0.0001*
Somatosensory (FaceTouch > FaceAlone)						
R S1	58	−18	32	4.63	20	0.0006**
L S1	−60	−26	16	11.32	929	<0.0001**
	−66	−20	22	8.67		
L S2	−50	−32	22	14.84	6604	<0.0001*
R posterior parietal	24	−40	62	7.27	1231	<0.0001*
L posterior parietal	−24	−42	68	7.31		
R temporoparietal junction (BA39)	54	−64	10	10.08	7543	<0.0001*
L temporoparietal junction (BA39)	−52	−56	10	12.48	6604	<0.0001*
L precentral gyrus (BA6)	−38	−4	48	5.38	28	0.0036*
R insula	40	−12	−6	11.08	7543	<0.0001*
L insula	−36	18	2	5.02	15	0.0089*
R cuneus (BA7)	12	−78	34	6.86	1637	<0.0001*
L calcarine sulcus	−18	−66	8	6.58		
L posterior parietal area (BA7)	−6	−48	52	6.63	1231	<0.0001*
L mid cingulate (BA31)	−12	−26	40	6.73	135	<0.0001*
R mid cingulate (BA31)	4	−4	38	5.76	230	<0.0001*
Visual (FaceVis > FaceAlone)						
R calcarine sulcus	12	−84	0	9.67	4115	<0.0001*
R lateral occipital (V2)	38	−80	18	9.75		
L medial lingual gyrus V1 / V2	−10	−84	−2	10.07	17137	<0.0001*
L superior parietal (BA7)	−22	−64	50	5.27	23	0.0050*

Note: All coordinates reported in MNI space. *Indicates corrected for the whole brain volume. **Indicates *P* < 0.05 corrected for small volume based on predefined ROIs using masks from standard atlases (see Materials and Methods section). L, left; R, right; BA, Brodmann area.

**Table 3:** Processing of task-irrelevant stimuli across all 3 modalities (conjunction: auditory, somatosensory, and visual) versus the FaceAlone condition

Area	*xyz* coordinates			*T* value	size (voxels)	*P* value
R temporoparietal junction	52	−52	8	4.17	1044	<0.0001*
L temporoparietal junction	−54	−62	10	4.47	990	<0.0001*
R cuneus	22	−68	24	3.76	3887	<0.0001*
R posterior cingulate	6	−30	26	1.93		
L mid cingulate	−12	−24	40	2.74		
R premotor	50	6	40	2.34	483	<0.0001*

Note: All coordinates reported in MNI space. *Indicates corrected for the whole brain volume. L, left; R, right.

**Table 4:** Modulation of task-irrelevant stimulus processing during emotion

Area	*xyz* coordinates			*T* value	Size (voxels)	*P* value
Auditory enhancement (Aud NEG > Aud NEU) with habituation effects in time						
R PAC (Heschl's gyrus)						
R Te1.0	52	−6	−4	3.19		0.0353**
R Te1.2	52	−2	−4	3.05		0.0285**
L PAC (Heschl's gyrus)						
L Te1.2	−56	−6	−4	3.01		0.0509**
Somatosensory enhancement (Touch NEG > Touch NEU)						
R S1	10	−36	72	4.14		0.0235**
L S1	−58	−16	34	4.08	507	0.0019*
R S2	38	−24	16	3.6	286	0.0256*
	62	−2	6	4.11	687	0.0003*
R insula	42	0	4	4.54		
Visual decrease (Vis NEU > Vis NEG)						
L calcarine sulcus	−16	−100	−6	4.2		0.0128**

Note: All coordinates reported in MNI space. *Indicates corrected for the whole brain volume. **Indicates *P* < 0.05 corrected for small volume based on predefined ROIs using masks from standard atlases (see Materials and Methods section). L, left; R, right; BA, Brodmann area.

**Table 5:** Modulation of task-irrelevant stimulus processing during emotion: conjunction of the “increase” effects (NEG > NEU) in auditory and somatosensory conditions

Area	*xyz* coordinates			*T* value	Size (voxels)	*P* value
R inferior frontal (p.triangularis)	48	20	−18	2.87	485	0.0003*
R anterior cingulate	4	20	20	2.83	301	0.0053*
L anterior insula	−40	0	−6	2.72	198	0.0337*
L orbitofrontal	−44	16	−6	2.37		
L posterior cingulate	−2	−42	24	2.56	191	0.0386*
R mid cingulate	2	−26	30	2.39		

Note: All coordinates reported in MNI space. *Indicates *P* < 0.05 corrected for whole brain volume.

### Task-Irrelevant Stimulus Processing

**Figure 2.**
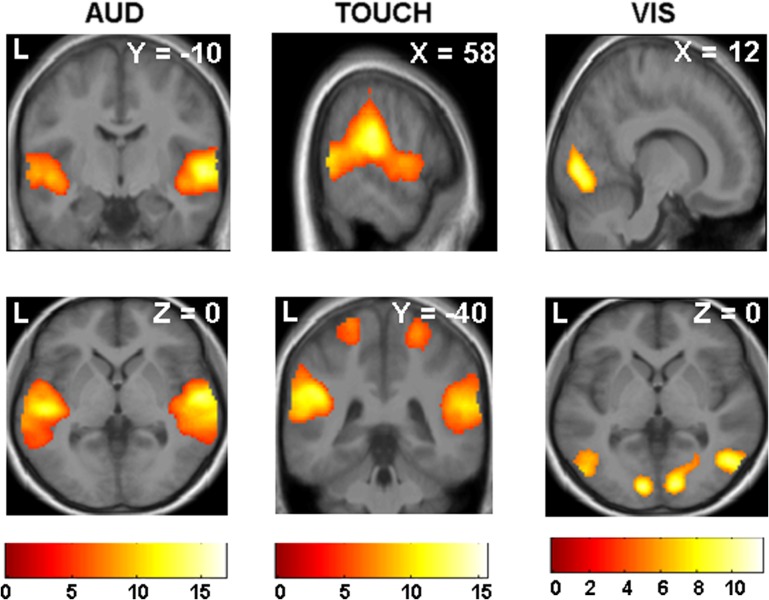
Processing of task-irrelevant stimuli across emotion conditions. Main BOLD activations for all FaceAud versus FaceAlone trials (left) were located in bilateral Heschl's gyrus and higher auditory regions. Main BOLD activations for all FaceTouch versus FaceAlone trials (middle) were located in bilateral S1 and left S2. Main BOLD activations for all FaceVis versus FaceAlone trials (right) were located in bilateral visual cortex. The plane coordinates of each slice are indicated in the upper right-hand corner. Bright colors represent significance levels of contrasts, as indicated by the scale bars (*T* values). BOLD responses are rendered on an average anatomical image from all participants.

Before examining emotional modulation of auditory, tactile, and visual processing, we isolated sensory responses to each sensory modality by contrasting trials with task-irrelevant stimulation from those in which faces were processed alone (e.g., [FaceAudNEG + FaceAudNEU] > [FaceAloneNEG + FaceAloneNEU], hereafter FaceAud > FaceAlone, Fig. 2, Table 2). As expected, auditory stimuli evoked bilateral activations in PAC, secondary auditory cortex (BA42 and planum temporale/BA22), as well as medial geniculate nucleus and precuneus. Somatosensory stimuli (FaceTouch > FaceAlone) activated bilateral S1, left S2, bilateral posterior parietal cortex, and bilateral posterior insula. Finally, visual processing (FaceVis > FaceAlone) recruited bilateral primary and secondary visual areas. A conjunction analysis of these 3 contrasts revealed common activations for all sensory modalities in bilateral temporoparietal junction, premotor, and cingulate cortices (Table 3). This network is known to be involved in multimodal attentional orienting toward auditory, visual, and tactile changes in the environment (Downar et al. 2000, 2001, 2002; Schwartz et al. 2005).

### Emotional Face Processing

To determine the emotional impact of facial expressions, we compared the FaceAloneNEG versus FaceAloneNEU trials. Increased responses were observed in right dorsolateral prefrontal cortex (*x* = 26, *y* = 22, *z* = 56, cluster size: 176 voxels, *T* value: 4.98, *P* = 0.047, corrected for whole brain volume). No other clusters survived our strict thresholds in this contrast, even when considering habituation effects by using a parametric time regressor. Given its well-known involvement in emotional and gain modulation of sensory processing (see Pourtois et al. 2012, for review), we also inspected bilateral amygdala ROIs but found only weak increases in the right side (*x* = 22, *y* = −14, *z* = −24; cluster size: 6 voxels, *T* value: 1.75, *P* = 0.04, uncorrected), which habituated over time. Although below significance threshold, this effect is in the expected direction (LeDoux 2000; Vuilleumier 2005) and is likely to be weak due to the many repetitions of face stimuli across all trials, causing a loss of signal over time in both conditions (Breiter et al. 1996; Sergerie et al. 2008). In this vein, examining the parametric time regressors of the conditions FaceAloneNEG and FaceAloneNEU separatedly revealed a marked habituation effect over the course of the experiment along the bilateral temporal lobes, including the right fusiform for both the NEU (*x* = 44, *y* = −44, *z* = −14, cluster size: 252 voxels, *T* value: 4.53, *P* = 0.049, corrected for whole brain volume) and the NEG conditions (*x* = 42, *y* = −42, *z* = −16, cluster size: 311 voxels, *T* value: 4.76, *P* = 0.024, corrected for whole brain volume). The fusiform cortex is, again, a face-processing region with well-known responsiveness to emotion in faces (Vuilleumier and Pourtois 2007).

Finally, a parametric analysis including pupil size and the interaction between pupil size and trial order for this contrast revealed no significant effects.

### Modulation of Task-Irrelevant Stimulus Processing by Visual Emotion

**Figure 3.**
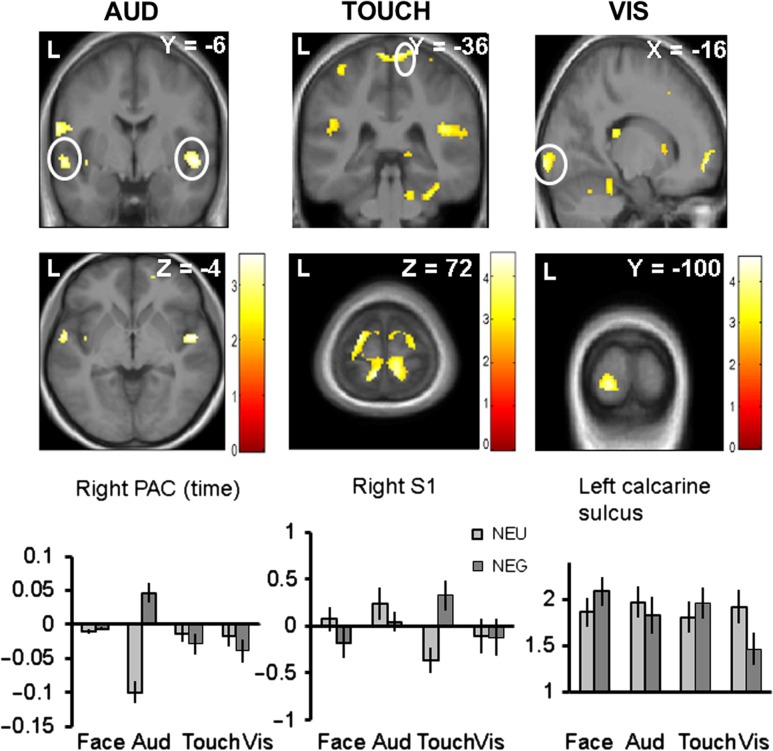
Emotion effects on sensory responses. Left: auditory processing (FaceAud > FaceAlone with fearful faces) > (FaceAud > FaceAlone with neutral faces). Bilateral activations in  PAC (upper panel), representing selective increase to tones during fearful faces which habituated over time, together with the plot of time-dependent responses (±SEM, bottom panel) across conditions for the right PAC peak (mean *x y z*, 52 −6 −4; *P* < 0.005 for illustration). Negative values reflect greater habituation for the NEU (neutral) compared with the NEG (fearful) trials. Middle: somatosensory processing (FaceTouch > FaceAlone with fearful faces) > (FaceTouch > FaceAlone with neutral faces). Bilateral increases in primary somatosensory cortex (S1) during fearful faces and average activity (±SEM, bottom middle) across conditions for the right S1 peak (mean *x y z*, 10 −36 72; *P* < 0.005 for illustration). Right: visual processing. Decreased responses in left calcarine sulcus induced by emotion (FaceVis > FaceAlone with neutral faces) >  (FaceVis > FaceAlone with fearful faces) and average activity (±SEM, bottom right) across conditions for the left calcarine sulcus peak (mean *x y z*, −16 −100 −6; *P* < 0.005 for illustration). Aud: FaceAud condition; Touch: FaceTouch condition; Vis: FaceVis condition. The plane coordinates of each slice are indicated in the upper right-hand corner. Bright colors represent significance levels of contrasts, as indicated by the scale bars (*T* values). BOLD responses are rendered on an average anatomical image from all participants.

We next tested for emotional modulation of sensory responses to concomitant stimuli across the 3 modalities. As shown in Figure 3 and Table 4, auditory brain responses to task-irrelevant tones were enhanced in the NEG condition, relative to NEU, in bilateral PAC. These effects became apparent only when testing for habituation effects through the parametric time modulator (with contrasts on the parametric regressors). No effect was associated with the inverse contrast (FaceAudNEU > FaceAudNEG), even at the most liberal thresholds, irrespective of the inclusion of time habituation.

Likewise, the emotional NEG condition increased responses to the task-irrelevant tactile stimulation (FaceTouchNEG > FaceTouchNEU) in several somatosensory regions, including bilateral S1 (likely corresponding to the face somatosensory area), right S2, and right insula. This increase was found in the main contrast between conditions, with no significant habituation in the parametric analysis. No effect was observed with the inverse contrast (FaceTouchNEU > FaceTouchNEG).

In turn, visual responses to task-irrelevant checkerboards showed a marked decrease in left V1 (FaceVisNEU > FaceVisNEG, Fig. 3, Table 4). Thus, unlike responses to sound and touches, visual activation to peripheral flickers was reduced in the presence of fearful face stimuli at fixation.

It should be mentioned that a weak effect of increase, which did not survive correction thresholds, was also observed in the right hemisphere (in the opposite contrast FaceVisNEG > FaceVisNEU), characterized by a cluster of 46 voxels that extended to V1 (*x* = 24, *y* = −92, *z* = 0; *T* value: 3.46, *P* value = 0.0004 uncorrected for whole brain volume) and lateral occipital gyrus (*x* = 32, *y* = −94, *z* = 8; *T* value: 3.55, *P* value: 0.0003, uncorrected). Moreover, this V1 increase became apparent only when including time habituation as a parametric modulator (with contrasts on the parametric regressors). Because this enhancement did not survive correction for the whole brain or V1 ROI volume, it will not be further considered (see Discussion); but we note in passing that such dissociation between the left decrease and right (weak) increase might originate in well-known hemispheric asymmetries associated with both vision and emotion processing (Heinze et al. 1998; Evans et al. 2000; Peyrin et al. 2005; Musel et al. 2013), which suggest left-hemispheric advantage for processing stimulus information at the local level (possibly reduced by emotion signals), but right-hemispheric advantage for processing more global information (possibly enhanced by emotion).

Finally, additional analyses to examine the parametric effects of pupil size, as well as of the interaction between pupil and trial order on all contrasts of interest, revealed no significant voxels in any of the main effects. Only 2 weak (and nonsignificant) modulations by pupil size alone were observed for the contrasts FaceTouchNEG > FaceTouchNEU (Right S1: *x* = 58, *y* = −10, *z* = 40; *P* value: 0.0837; small-volume corrected; *T* value: 3.84) and FaceVisNEU>FaceVisNEG (Right V1: *x* = 14, *y* = −90, *z* = 2; *P* value: 0.0016; uncorrected; *T* value: 3.84), which accord with results from the main analyses.

We also performed additional control analyses to confirm a genuinely specific effect of visual emotion processing on sensory ROIs, and rule out a more general modulation of all sensory cortices irrespective of sensory inputs (see the Materials and Methods section). These analyses revealed no changes of BOLD responses in V1 or in S1 during auditory stimulation (contrast FaceAudNEG > FaceAudNEU), either for the main effect of emotion condition regressors or for the contrast on parametric regressors that accounted for habituation in time. Similarly, the contrast FaceVisNEU > FaceVisNEG showed no modulation of PAC or S1 during visual stimulation. Again, no effects were observed for the parametric habituation regressors.

Lastly, there were no modulatory effects on V1 during touches (contrast FaceTouchNEG > FaceTouchNEU), but we observed a significant modulation on the peak *x* = 62, *y* = −2, *z* = 4 (*P* value: 0.0135, small-volume corrected; *T* value: 4.02) when using the PAC mask. This could reflect a partial overlap of the PAC ROIs with S2 and thus represent somatosensory responses in S2 misattributed to auditory areas due to anatomical proximity, or artifactual auditory stimulation due to the tactile device (see Discussion section).

### Multimodal Effects of Emotion

A conjunction analysis across all 3 sensory modalities was tested for any common pattern of activity elicited by the presence of emotional cues. This conjunction (performed after masking with relevant main effects; see Materials and methods) did not show any common effects surviving correction thresholds. Again, no common modulations were observed when including the contrast FaceVisNEU > FaceVisNEG (given the decrease observed for the Visual condition), instead of FaceVisNEG > FaceVisNEU.

**Figure 4.**
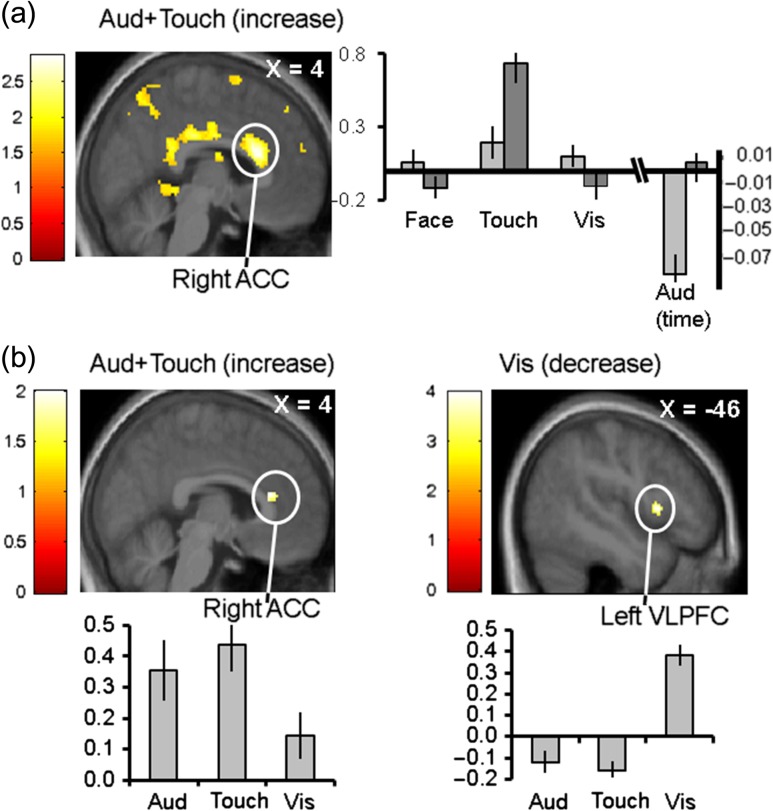
Conjunction effects and functional connectivity. (*a*) Conjunction analysis of the main emotion effects for the Auditory and Touch conditions (NEG > NEU), the 2 sensory modalities showing an increase in the modality-specific contrasts. Average neural activity (±SEM) is plotted (right panel) across conditions for the right anterior cingulate cortex (ACC) peak (mean *x y z*, 4 20 20; *P* < 0.005 for illustration). The auditory condition is plotted using a different scale to illustrate the time-dependent responses (see Fig. 3 and Materials and Methods), which reflect habituation effects observed in this modality (i.e., showing greater habituation for NEU compared with NEG trials). (*b*) PPI maps identifying brain regions functionally connected to the sensory areas that exhibited increased response in the emotional condition (Auditory and Touch; left panel), or decreased response during the emotional condition (Visual; right panel). PPI parameters (±SEM, bottom panel) are plotted across conditions for the right ACC peak (*x y z*, 4 30 14; *P* < 0.005 for illustration) that correlated with sensory increases (Audition and Touch), and for the left VLPFC peak (*x y z*, −46 12 6; *P* < 0.005 for illustration) that correlated with sensory decreases (Vision). Both PPIs were calculated using those left-hemisphere seeds in primary sensory areas that showed the strongest peaks of emotion effects (contrasts NEG > NEU for FaceAud and FaceTouch but NEU > NEG for the FaceVis condition). The functional contrasts used as psychological variables for PPI computation where NEG > NEU in all 3 sensory conditions, but considering the negative correlation effects for the visual seed. Coordinates of the depicted brain slice are indicated in the upper right-hand corner. Bright colors represent significance levels of contrasts, as indicated by the scale bars (*T* values). BOLD responses are rendered on an average anatomical image from all participants.

A second conjunction was then performed, restricted to the auditory and the tactile conditions, as only these 2 sensory modalities were positively modulated by the concurrent presentation of emotional faces (whereas vision exhibited a negative modulation). By applying the same approach as in the 3-way conjunction (using the same SPM model), this analysis demonstrated shared activations in the anterior, posterior, and middle cingulate cortices, as well as in the left anterior insula, left orbitofrontal cortex (OFC) and right inferior frontal gyrus (Table 5; Fig. 4*a*).

### Functional Connectivity

To test for possible sources of modulations influencing sensory responses in the presence of emotional cues, we computed PPI maps across the 3 sensory modalities, using the primary sensory regions modulated by emotional facial expressions (e.g., PAC during auditory stimulation) as seeds, and the functional contrast mapping each of these regions (e.g., FaceAudNEG > FaceAudNEU) as psychological variables. The seed regions chosen for PPI analysis were the voxel peaks showing the maximum *T* value on the left hemisphere, given that the effects on the visual modality were left-lateralized. This analysis led to 3 independent connectivity maps, revealing the region mostly coupled with PAC (positive correlation), S1 (positive correlation), and V1 (negative correlation, given the decrease observed in the main contrasts), when auditory, somatosensory, and visual information were presented concurrently with a negative (relative to neutral) facial expression (FaceAudNEG > FaceAudNEU, FaceTouchNEG > FaceTouchNEU and FaceVisNEG > FaceVisNEU). A conjunction analysis between these 3 maps did not reveal any significant voxels. Then, for consistency purposes, we applied the same logic as for the conjunction analysis of the main effects, described above, by testing PPI parameters only from the 2 sensory modalities associated with emotional “enhancement” (i.e., FaceAudNEG > FaceAudNEU ∩ FaceTouchNEG > FaceTouchNEU). This analysis revealed that PAC (for audition) and S1 (for touch) exhibited a common increase in functional connectivity with a portion of the anterior cingulate cortex (ACC) (*x* = 4, *y* = 30, *z* = 14; peak *P* value, uncorrected: 0.00064; *T* value: 2.00; cluster size: 49; Fig. 4*b*), overlapping with the region already implicated in the conjunction of the main effects. This shared connectivity effect may reflect modulatory emotion signals that boosted responses in the auditory and the somatosensory cortices for the Auditory and the Touch conditions, respectively. On the other hand, we analyzed separately the PPI parameters from the visual modality, which revealed selective “decrease” of functional connectivity in the left posterior ventrolateral prefrontal cortex (VLPFC; *x* = −46, *y* = 12, *z* = 6; peak *P* value, uncorrected: 0.000102; *T* value: 3.98; cluster size: 18; Fig. 4*b*).

## Discussion

We provide, for the first time, evidence that visual emotional events can alter neural responsiveness of early sensory cortices to simultaneous stimuli across multiple sensory modalities. By presenting unpredictable and task-irrelevant stimuli from 3 different modalities (auditory, tactile, or visual), while participants directed attention to faces, we could define reliable sensory-specific responses in auditory cortices for tones, somatosensory cortices for touches, and occipital visual areas for checkerboards. Critically, the comparison of these responses in the presence of fearful versus neutral faces revealed significant emotional modulations for all modalities. Cortical activations to both auditory and tactile stimuli were bilaterally enhanced in the emotional relative to the neutral condition. Specifically, auditory responses were increased in bilateral PAC. Likewise, tactile responses were increased in bilateral S1, right S2, and right insula. No decrease was observed for these 2 modalities, indicating heightened sensory reactivity due to emotional signals, with no apparent cost due to competition with face processing.

In contrast, visual responses showed a decrease in the left calcarine sulcus. Such decreases were not observed in other modalities. The latter effect may therefore reflect a mechanism of sensory competition within the visual modality, such that the emotional faces captured more attentional resources in the visual field at the cost of concurrent peripheral information (Ciaramitaro et al. 2007; Keil et al. 2007). Several studies have described reduced activations to peripheral distractors when participants perform a central visual task with higher processing demands (Rees et al. 1997; Schwartz et al. 2005), or reduced electrophysiological responses when visual onsets are superimposed on emotionally arousing compared with neutral pictures (Muller et al. 2008). It is worth taking into account, however, that the right calcarine sulcus showed weak (and nonsignificant) increased responses in the emotional relative to the neutral condition. Even though this effect did not survive correction thresholds, it may be indicative of a hemispheric dissociation between global (right-lateralized) and local (left-lateralized) processing when stimuli compete within a sensory channel. Further studies should examine this potential 2-fold effect more in detail.

Please note that the emotional facial expressions modulated sensory-specific cortices always during the presence of stimuli of the corresponding modality. No systematic modulation of sensory-specific cortices was observed during the processing of stimuli from a different modality, with the only exception of a peak corresponding to the lateral portion of right PAC (*x* = 62, *z* = −2, *y* = 4) during the touch condition. One explanation might be that the tactile stimulation device produced a weak click sound, which might have been detected by some subjects over the scanner noise. However, based on the probabilistic maps used for our ROI analyses (Eickhoff et al. 2005, 2007), this peak could be attributed not only to the right PAC (i.e., Te1.2) with a probability of 40%, but also to the right S2, with an equal probability of 40%. Therefore, although we cannot fully rule out an additional modulation of auditory cortex, we surmise that the most suitable explanation of our data is that of a true bisensory influence (i.e., visual emotion cues impacted sensory-specific cortices in the presence of stimuli from the corresponding modality), rather than a more global and unspecific boosting of sensory responses across all cortical areas during visual emotion processing. Moreover, as clearly visible in Figure 3, none of the primary sensory clusters exhibiting a modulation for stimuli of the corresponding modality showed a modulation elicited by stimuli from a different modality.

In sum, these results confirm and extend current models of crossmodal attention, which posit that attentional processes modulate early modality-specific neural responses across the visual, auditory, and somatosensory cortices (Calvert 2001; Eimer and Driver 2001; McDonald et al. 2003; Busse et al. 2005; Spence 2010; Wesslein et al. 2014). We suggest that emotion processing may exert similar modulatory effects across sensory modalities through modulations of attentional systems.

Taken together, our findings raise several important questions for future research. First, whether similar effects occur both crossmodally and intramodally when emotion signals are conveyed through audition or touch. Second, whether these effects depend on the temporal relationship between the emotion signals and the concomitant sensory inputs. In our study, the onset times of task-irrelevant stimuli were based on previous electroencephalography (EEG) research that described an optimal window for visual-auditory emotional influence (Selinger et al. 2013). Therefore, these crossmodal emotional effects reported here might be restricted to a particularly short (e.g., 100–150 ms) temporal asynchrony. Similarly, spatial attention can operate across different sensory modalities (Driver and Spence 1998; Eimer 2001) in a time-dependent manner (Spence and Driver 1998; Spence et al. 1998; Van der Stoep et al. 2015). Such temporal dependence may also exist for emotional effects (e.g., Bradley et al. 2006; Muller et al. 2008) and could explain, for instance, why some EEG studies reported a reduction of neural responses to auditory startling (Schupp et al. 1997; Cuthbert et al. 1998; Keil et al. 2007) or tactile stimulation (Montoya and Sitges 2006) during affective picture processing. In addition, our findings could have been facilitated by the spatial proximity between stimuli (i.e., near the subjects’ head), a factor also known to influence crossmodal attention (Driver and Spence 1998).

Our results extend previous research which showed that visualizing emotional pictures, relative to neutral, may lead to stronger electrophysiological responses to unattended sounds (Sugimoto et al. 2007; Domínguez-Borràs et al. 2008a, 2008b, 2009; Garcia-Garcia et al. 2010; Selinger et al. 2013), reduce background noise in brainstem responses (Wang et al. 2010), facilitate spatial orienting toward tactile stimuli (Poliakoff et al. 2007), or enhance contrast sensitivity for subsequent visual targets (Phelps et al. 2006). Similarly, expecting high pain results into higher ratings of incoming unpleasant odors and highly unpleasant-odor expectations increase the ratings of subsequent pain stimulation (Sharvit et al. 2015). Here, we show for the first time that emotional modulations of sensory processing can occur simultaneously in early sensory cortices across multiple modalities, even for task-irrelevant and unpredictable stimuli. Note that some of these effects were apparent only when habituation effects were considered for analysis, suggesting that these modulations faded over the experimental session. Such habituation is frequently observed with the repetition of emotional stimuli over successive trials (Plichta et al. 2014).

Note that we can only assume that the task-irrelevant stimuli were indeed unattended to the extent that participants had to simultaneously respond to the target faces, with accuracy in this task of nearly 100% (Escera et al. 1998; Parmentier et al. 2008; SanMiguel et al. 2010). However, due to the simplicity of the task demands, it is possible that task-irrelevant stimuli were partly or occasionally attended. In either case, to ensure adequate attentional focus on faces, we included only trials with correct behavioral responses in our analyses. Further, our critical comparisons always concerned the same task-irrelevant stimulus conditions (in 3 sensory modalities) in the different (emotion vs neutral) contexts, also ensuring that any degree of voluntary attention toward the distractors on some trials would occur similarly in both cases and allow us to compare the task-irrelevant sensory responses between different emotion contexts. Hence, any uncontrolled attention to distractors should not affect our main results.

Importantly, the design of our study also enabled us to identify brain regions commonly recruited during emotional modulations across the different sensory modalities. Conjunction analysis for conditions with enhanced responses (auditory and somatosensory) revealed selective increases in several limbic areas including ACC, posterior and middle cingulate cortices, insula, and OFC as well as inferior frontal gyrus, all recruited more strongly during the negative than neutral conditions, reflecting a shared activity pattern concomitant to the enhancement of auditory and somatosensory cortices. Similarly, a conjunction of functional connectivity changes during emotional modulations across these 2 sensory modalities revealed shared coupling with the rostral ACC. This cluster of differential connectivity overlapped with the region already implicated in the conjunction of the main effects. Distinct changes in functional connectivity were associated with the negative modulation of visual cortex, for which we observed selective effects in the left posterior VLPFC. ACC and OFC belong to a well-described network thought to influence perception and attention in response to emotional signals, possibly through direct connections with fronto-parietal areas and sensory cortices (Cavada et al. 2000; see Domínguez-Borràs et al. 2012; Domínguez-Borràs and Vuilleumier 2013). ACC is a key node of the salience detection network, implicated in various aspects of alertness and attention orienting (Seeley et al. 2007). Furthermore, the anterior insula has also been proposed to constitute a crucial hub for dynamic interactions between large-scale brain networks involved in externally oriented attention and internally oriented cognition (Menon and Uddin 2010). Its main function may be to mark salient events for additional processing and initiate the appropriate control signals (Menon and Uddin 2010). It has been suggested that this region, together with ACC, may form a salience system that segregates the most relevant among internal and extrapersonal stimuli in order to guide behavior, by facilitating rapid access to the motor system (Menon and Uddin 2010). In turn, VLPFC is a cortical region with an important role in emotion regulation (Ochsner and Gross 2005; Ray and Zald 2012), implicated in the controlled allocation of processing resources between competing (i.e., emotional and nonemotional) stimuli (Yamasaki et al. 2002; Fichtenholtz et al. 2004; Vuilleumier 2005). In sum, these results highlight the existence of a multimodal network for emotional control of perception, mediated by the cingulate, insula, and orbitofrontal cortices for sensory potentiation, and VLPFC for sensory inhibition.

Nevertheless, we draw attention to the fact that the conjunction analysis of main effects was implemented by building a specific model that included regressors representing the 3 contrasts through which we found the main modulatory effects. This model, which was exclusively used for testing conjunction, included the auditory (Aud) regressors accounting for parametric modulation of trial order (as this was the contrast showing the main modulatory effect for this modality) and the normal regressors for Touch and Vision. In contrast, the PPI conjunction effects were observed when including all the PPI normal regressors (including that for Aud). Since both conjunction analyses (i.e., main effects and PPI) yielded selective modulations in anatomically overlapping clusters in right ACC, we believe that both results are valid and comparable. Accordingly, we suggest that, whereas the modulatory effects of right ACC on auditory responses (i.e., for the FaceAudNEG > FaceAudNEU contrast) varied over the course of the experiment, the differential coupling between this region and PAC across emotional conditions kept a relatively stable pattern over time.

Note that in keeping with a causal role for emotional processing in modulating brain responses to sensory stimuli, fearful faces produced greater activation in right DLPFC (Yamasaki et al. 2002; Fichtenholtz et al. 2004; Ochsner and Gross 2005; Vuilleumier 2005; Ray and Zald 2012). Even though no significant modulations were observed in visual cortex or amygdala, as it would be expected according to the literature on emotional face processing (LeDoux 2000; Pessoa et al. 2002; Pourtois et al. 2004b, 2012; Vuilleumier 2005), 2 additional arguments support a reliable emotional impact of fearful face stimuli in our study. First, participants consistently rated fearful faces as more emotionally negative and alerting than neutral faces. Second, presenting fearful faces alone elicited differential pupil responses in relation to the neutral faces, an effect that habituated over the course of the experiment. This habituation, again, is not surprising given that faces were repeatedly presented (312 times) throughout the experimental session. Pupillary size is a reliable measure of autonomic arousal during emotion processing (Bradley et al. 2008; Kreibig 2010), providing indirect support to the notion that arousal may play a crucial role in the modulatory effects of emotion on sensory processing (Harrison et al. 2013). Note, however, that our fMRI analyses including parametric modulations of pupil size revealed no significant effects of this physiological value in any of the contrasts. One explanation could be the lack of statistical power due to the fact that only 15 subjects (those from whom we had usable pupil data) were considered for this analysis, instead of the 19 participants included in all other analyses not involving pupil size.

In conclusion, our results demonstrate that visual emotional processing modifies early sensory responses, not only within vision, but also across the auditory and somatosensory modalities. These effects appear to be controlled by supramodal networks for emotional regulation of perception and attention (Vuilleumier 2005; Domínguez-Borràs and Vuilleumier 2013), involving ACC, insula, and OFC for sensory response potentiation, and VLPFC for sensory response inhibition. Thus, sensory events that would be irrelevant in affectively neutral conditions may become more salient in a potentially threatening environment, as long as these events do not compete within a sensory channel. Our results provide novel support to the notion that threatening contexts may indiscriminately sensitize reactivity to all incoming sensory stimuli, perhaps at a loss of stimulus specificity in some conditions (Baas et al. 2006; Cornwell et al. 2007; Dunning et al. 2013). However, we also provide evidence for the opposite effects when sensory competition occurs.

In this respect, it is important to point out that our results only demonstrate that neural activity in primary sensory regions was modulated by emotion contexts, a phenomenon that is broadly consistent with other modulatory effects uncovered by past research on attention (Hillyard et al. 1973; Posner 1980; Desimone and Duncan 1995; Hillyard and Anllo-Vento 1998; Kastner et al. 1998; Kastner and Ungerleider 2001; Rousselet et al. 2004; Serences et al. 2004; Cavanagh and Alvarez 2005; Naatanen et al. 2011) and emotion (Lang et al. 1998; Morris et al. 1998; Vuilleumier et al. 2001; Pessoa et al. 2002; Pourtois et al. 2004a; Grandjean et al. 2005; Sabatinelli et al. 2005; Ethofer et al. 2011; Keil et al. 2011). Such effects have typically been related to enhanced sensory processing, but here we did not directly assess perceptual performance toward the task-irrelevant stimulation (e.g., by means of psychophysics). Therefore, we cannot ensure that the observed increase/decrease in sensory regions reflect per se a deeper/shallower cortical processing of the stimuli. In support of deeper sensory processing, though, several attention studies have thoroughly examined the functional links between changes in neural activity and the corresponding behavioral (perceptual) advantage. For instance, BOLD responses in Heschl's gyrus were found to correlate positively with sound discriminability and identification in perceptual tasks (and not simply with RT or improved detection; see Binder et al. 2004), or to reflect sensitization and lower hearing thresholds to sounds (Reznik et al. 2014). In the visual domain, attentional gain of BOLD sensory signals was formally linked to improved performance in perception tasks (Liu et al. 2005; Pestilli et al. 2011; see also Serences 2011). Nonetheless, increased neural response alone may not be sufficient to explain attention-induced changes in perceptual performance (Pestilli et al. 2011; Serences 2011). In any case, sensory modulations observed in our study did not arise from differences in physical features within the task-irrelevant stimulation, as stimuli were identical across emotional conditions; hence they reflect a true modulation of sensory input within the early cortical pathways.

Note that another example of transmodal effects of emotion may be the classic “startle potentiation” phenomenon (originally described by Brown et al. 1951), where brief muscle contractions evoked by sudden acoustic, visual, or tactile stimuli (i.e., startle reflex; Koch 1999) are potentiated in fear contexts (Koch 1999; Grillon and Baas 2003). In humans, this phenomenon has been demonstrated during the visualization of emotionally laden pictures, which typically induces stronger eye-blinks in response to sudden, task-irrelevant acoustic stimuli, as compared with the visualization of neutral pictures (e.g., Lang 1995; Stanley and Knight 2004; Bradley et al. 2006; Dunning et al. 2013). Our results may additionally add to our current knowledge of this and other related emotion phenomena. While the role of a motor facilitation has been proposed to explain the enhanced startle response (Koch 1999), our results suggest that sensory enhancement also occurs and might also contribute, or at least accompany, this process. However, startle potentiation should be compared with extreme caution and cannot be reduced to enhanced perceptual processing, as it is known to be partly subserved by specific neural mechanisms in the brainstem (Baas et al. 2006). Altogether, these findings may also help understand sensory disturbances in psychiatric conditions, such as in post-traumatic stress disorder, where exaggerated startle response is a diagnostic criterion (DSM-V, 2013, A.P.A.), or attentional biases in anxiety disorders (Grupe and Nitschke 2013).
